# A Nomogram to Predict Recurrence-Free Survival Following Surgery for Vestibular Schwannoma

**DOI:** 10.3389/fonc.2022.838112

**Published:** 2022-04-28

**Authors:** Zehan Zhang, Ding Zhang, Xudong Shi, Bingyan Tao, Yuyang Liu, Jun Zhang

**Affiliations:** ^1^Medical School of Chinese People’s Liberation Army (PLA), Beijing, China; ^2^Department of Neurosurgery, The First Medical Centre, Chinese People's Liberation Army General Hospital, Beijing, China

**Keywords:** vestibular schwannoma, nomogram, recurrence-free survival, Ki-67, web-based calculator, decision curve analysis

## Abstract

**Background:**

Vestibular schwannoma (VS) is the most common benign tumor of the posterior fossa. The recurrence of VS has always received widespread attention. This study aimed to develop a nomogram to predict Recurrence-free survival (RFS) following resection of VS.

**Methods:**

A total of 425 patients with VS who underwent resection at the Department of Neurosurgery in Chinese PLA General Hospital between January 2014 and December 2020 were enrolled in this retrospective study. The medical records and follow-up data were collected. Cox regression analysis was used to screen prognostic factors and construct the nomogram. The predictive accuracy and clinical benefits of the nomogram were validated using the area under the curve (AUC), calibration curves, and decision curve analysis (DCA).

**Results:**

The Cox regression analysis revealed that age (HR = 0.96; 95% CI 0.94 - 0.99; p < 0.01), EOR (HR = 4.65; 95% CI 2.22 - 9.74; p < 0.001), and Ki-67 (HR = 1.16; 95% CI 1.09 - 1.23; p < 0.001) were all significantly correlated with recurrence, and they were finally included in the nomogram model. The concordance index of the nomogram was 0.86. The areas under the curve (AUCs) of the nomogram model of 3-, 4- and 5-year were 0.912, 0.865, and 0.809, respectively. A well-fitted calibration curve was also generated for the nomogram model. The DCA curves also indicated that the nomogram model had satisfactory clinical utility compared to the single indicators.

**Conclusions:**

We developed a nomogram that has high accuracy in predicting RFS in patients after resection of VS. All of the included prognostic factors are easy to obtain. The nomogram can improve the postoperative management of patients and assist clinicians in individualized clinical treatment. Furthermore, we generated a web-based calculator to facilitate clinical application: https://abc123-123.shinyapps.io/VS-RFS/.

## Introduction

Vestibular schwannoma is the most common benign tumor of the posterior fossa (1). In the past 30 years, the widespread usage of magnetic resonance imaging has increased the number of patients diagnosed annually (2, 3), the rates among all ages ranged between 3.0 and 5.2 per 100,000 person-years (4). Common treatment options include observation, surgery, and radiation therapy, and surgery is the only way to remove the tumor (5). The primary goal of the treatment of vestibular schwannoma is to achieve long-term progression-free survival without significant neurological dysfunction.

The recurrence of vestibular schwannoma has always received widespread attention. A long-term investigation of tumor recurrence after surgery showed that the tumor control rate at 5, 10, 15, and 20 years after surgery was 93%, 78%, 68%, and 51%, respectively (6). Total tumor resection is the treatment goal of VS. However, considering the protection of patients’ neurological function, this goal cannot always be achieved, which increases the risk of tumor recurrence to some extent. Some studies have achieved better tumor control by radiotherapy for patients with residual tumors. However, further research is needed to determine whether all patients can benefit from radiotherapy, especially those at low risk of relapse. For a long time, we have lacked tools that can be used clinically to stratify patients at risk. An accurate prediction can help us detect tumor recurrence early and help identify patients who might benefit from adjuvant therapy.

Evidence suggests that the extent of resection and residual tumor size are risk factors for tumor recurrence (6–8). Besides, recent research shows that the Ki-67 index is closely associated with tumor recurrence (9, 10). Considering the limited accuracy and effectiveness of a single risk factor, a multi-factor comprehensive model may be more suitable for recurrence prediction. Mehdi et al. constructed a prediction model based on an artificial neural network and showed good performance in estimating recurrence probability (11). However, there is currently no nomogram model that can be used to predict the risk of recurrence of vestibular schwannoma after surgery.

The nomogram is an easy-to-use graphical model that enables users to calculate the probability of a clinical event in a single patient (12). In this study, we developed a nomogram based on the clinical and pathological characteristics of patients to predict the probability of postoperative recurrence, we also generated a user-friendly graphical interface, which will help clinical doctors to identify patients with a high risk of recurrence and personalized health care decisions.

## Method

### Study Population and Selection Criteria

Clinical and follow-up data of patients with VS who underwent surgical treatment at the Chinese PLA General Hospital from January 2014 to December 2020 were continuously acquired.

The inclusion criteria were as follows: (1) complete medical records including clinical, laboratory, imaging, and pathological information; (2) a diagnosis of VS confirmed by pathology; and (3) undergoing primary surgical treatment.

**Figure 1:**
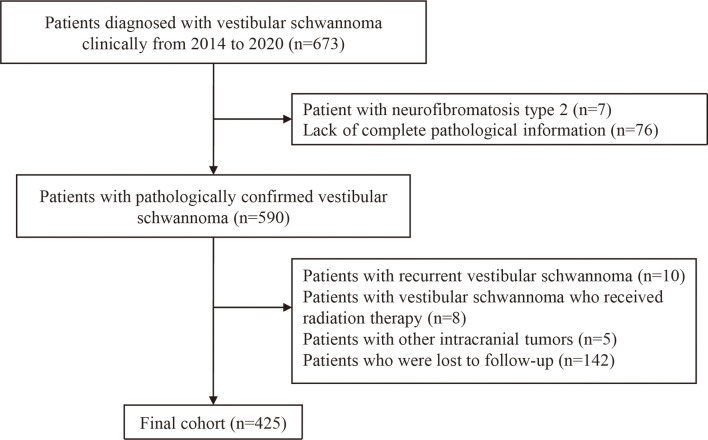
Flow chart of study population inclusion.

The exclusion criteria were as follows: (1) lack of complete pathology information; (2) patients with recurrent vestibular schwannoma (3) patients with NF2; (4) patients who have received radiation therapy; (5) patients with other intracranial tumors; and (6) loss to follow-up. A detailed data screening process is shown in **Figure 1**.

Demographic and clinical data were collected from the patients’ medical records, including age, sex, body mass index (BMI), tumor side, tumor size, Ki-67, the extent of tumor resection, and tumor recurrence. All patients undergoing surgical treatment underwent routine preoperative T1-weighted, T2-weighted, diffusion-weighted, and T1-weighted contrast-enhanced magnetic resonance imaging (MRI) sequences, and the maximum diameter of the tumor was measured and recorded.

Gross total resection (GTR) was defined as the total removal of the tumor as recorded in the surgeon’s operative note and no observable residual on postoperative enhanced MRI. Subtotal resection (STR) was defined as STR was defined as residual tumor of any degree, and patients were required to undergo enhanced MRI at 3 months and each subsequent year to assess tumor control. Patients were considered to have progressed if they required salvage treatment (surgery or radiosurgery) for clinical symptoms or tumor regrowth (>5 mm increase in the residual tumor size in the latest follow-up MRI) (9, 13).

### Statistical Analysis

Continuous variables were reflected as mean (standard deviation) or median (interquartile range) and were tested by Student’s t-test or Mann–Whitney U test. Categorical variables were reported as numbers with percentages and tested using the Chi-squared test or Fisher’s exact test. According to the Transparent Reporting of a multivariable prediction model of Individual Prognosis Or Diagnosis (TRIPOD) statement (14), the correlation matrix of all potential predictive factors was checked to avoid multicollinearity. To establish the nomogram, univariate and multivariate COX regression analyses were used to identify independent risk factors related to the recurrence of vestibular schwannoma. The optimal threshold of nomogram score for the probability of recurrence was ascertained by the Youden index and the closest-to-(0, 1) criterion. Survival curves were generated using the Kaplan–Meier method and compared using the log-rank test. The areas under the curve (AUC) of the receiver operating characteristic curve (ROC) and decision curve analysis (DCA) were applied to evaluate the performance of this nomogram. In order to evaluate the possible bias caused by missing follow-up, a sensitivity analysis was performed to assess differences in basic information between the final cohort and the lost follow-up patient cohort. All the P values <0.05 in two-sided was considered statistically significant. R software (version 4.0.2) with relevant packages was used for all statistical analyses and graphics. Several packages we used in the R environment include “survival”, “rms”, and “ggplot2”.

## Results

### Demographics and Characteristics of the Included Patients

A total of 425 patients who underwent surgery for VS met the criteria and were included in the analysis. The general characteristics of all patients were presented in **Table 1**. The mean age at diagnosis was 48.53 years; among the 425 cases, 176 patients were male and 249 patients were female; The mean tumor size was 2.89 cm. GTR was achieved in 283 cases and STR in 142 cases. The median Ki-67 index of the tumor was 5%. The median follow-up time was 37.0 (interquartile range, IQR, 24.0–53.0) months, and the overall recurrence rate was 8.1% (37 out of 425 subjects), and the estimated probability of 3-year RFS, 4-year RFS, and 5-year RFS were 93.5%, 89.6%, and 88.9%, respectively.

**Table 1:** Demographic information of the 425 patients.

Characteristics	Number
Age^#^	48.5 (12.1)
Sex^*^	
Female	249 (58.6%)
Male	176 (41.4%)
BMI^#^	24.4 (3.8)
Side^*^	
Left	204 (48.0%)
Right	221 (52.0%)
Tumor size^#^	2.9 (1.0)
Ki-67^†^	5.0 (2.0-5.0)
EOR^*^	
GTR	283 (66.6%)
STR	142 (33.4%)
Recurrence^*^	
No	388 (91.3%)
Yes	37 (8.7%)
3-year RFS (95% CI)	0.93 (0.91, 0.96)
4-year RFS (95% CI)	0.90 (0.86 0.94)
5-year RFS (95% CI)	0.89 (0.85, 0.93)
Median follow-up (months)^†^	37.0 (24.0-53.0)

*Data were expressed as number (%).

^#^Data were expressed as the means (± standard deviations).

^†^Data were expressed as the medians (interquartile ranges).

EOR, extent of resection.

Besides, of the 37 patients in this study who experienced tumor recurrence, 25 patients received postoperative radiotherapy, and the median time from surgery to need for salvage treatment was 35 (IQR, 23.0–49.0) months. Twelve patients underwent secondary surgery, and the median time from surgery to need for salvage treatment was 28.0 (23.8–47.5) months (**Table 2**).

**Table 2:** Type of salvage treatment and median time to salvage treatment in patients with tumor recurrence.

Treatment	Radiotherapy	Secondary operation	*p* value
N	25 (67.6%)	12 (32.4%)	
Time to salvage treatment^†^	35.0 (23.0-49.0)	28.0 (23.8-47.5)	0.987

^†^Data were expressed as the medians (interquartile ranges).

Sensitivity analysis showed no significant differences in age, sex, tumor side, tumor size, extent of surgical resection, BMI, and Ki-67 between the final cohort and the lost follow-up patient cohort (**Supplementary Table 1**).

### Identification and Selection of Prognostic Factors

We performed Cox regression analysis to identify significant prognostic factors correlated with tumor recurrence. As shown in **Table 3**, univariate regression analysis revealed that age, EOR, and Ki-67, were prognostic risk factors (p<0.001). To compare the discrimination between a single-variable model and a multi-variable combined model, we compared the C-indexes of age, EOR, Ki-67, and the three-factor combined model. The C-index of the three-factor combined model was 0.86, which was superior to that of age (C-index, 0.66, *p*<0.001), EOR (C-index, 0.69, *p*<0.001), and Ki-67 (C-index, 0.79, *p <*0.05). Therefore, we finally developed a model composed of age, EOR, and Ki-67. The detailed multivariate Cox regression results are shown in **Table 3**.

**Table 3:** Univariate and Multivariate Cox analysis of prognostic factors.

Exposure	Univariable analysis		Multivariable analysis		
	Hazard ratio (95%CI)	*p* value	Hazard ratio (95%CI)		*p* value
Age	0.95 (0.93, 0.98)	0.0002	0.96 (0.94, 0.99)		0.0023
Sex					
Female	Reference		–		
Male	0.88 (0.45, 1.74)	0.7235	–		
BMI	0.99 (0.91, 1.08)	0.7669	–		
Side					
Left	Reference		–		
Right	1.10 (0.57, 2.13)	0.7724	–		
Tumor size	1.03 (0.75, 1.41)	0.8613	–		
EOR					
GTR	Reference		Reference		
STR	3.87 (1.86, 8.06)	0.0003	4.65 (2.22, 9.74)		<0.0001
Ki-67	1.19 (1.12, 1.26)	<0.0001	1.16 (1.09, 1.23)		<0.0001

EOR, extent of resection; GTR, gross total resection; STR, subtotal resection.

### Development of the Nomogram Predicting Recurrence-Free Survival

**Figure 2:**
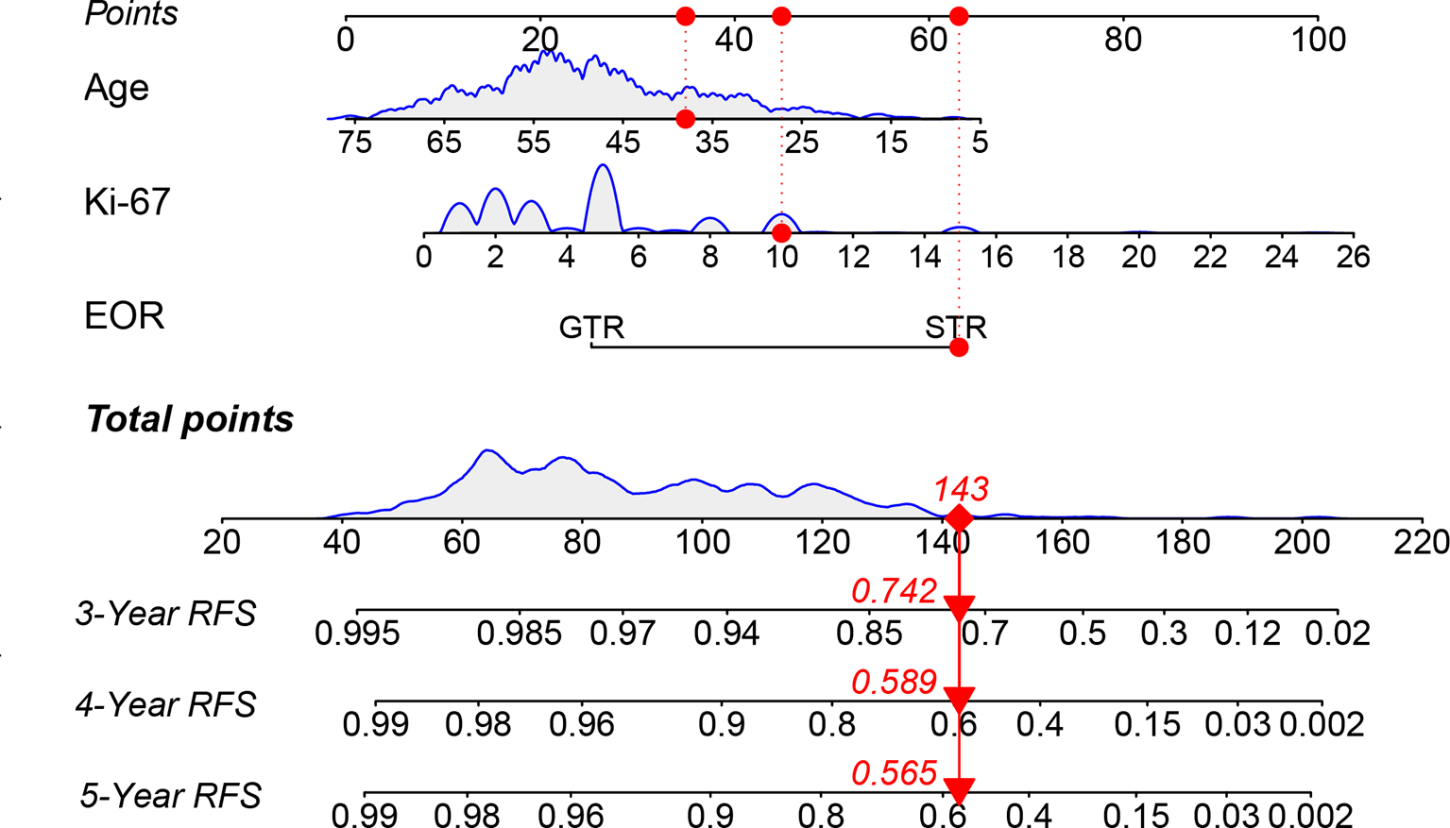
The nomogram predicting RFS of patients with vestibular schwannoma. RFS, recurrence-free survival; EOR, extent of resection; GTR: gross total resection; STR, subtotal resection.

Nomograms predicting 3-, 4-, and 5-year tumor recurrence-free intervals, incorporating all previously identified prognostic factors were constructed (**Figure 2**). The nomogram showed high predictive accuracy for RFS. The C-index of the nomogram was 0.860 (95% CI 0.801–0.918), and it had better predictive capability than other risk factors. By drawing lines on the nomogram, the corresponding points can be identified and added to obtain a total score. This score then corresponds to an individual estimate of a 3-, 4- and 5-year recurrence-free percentage. As an example, the red marker symbol showed the nomogram used for a 38-year-old patient with STR of VS, the pathology report showed that the Ki-67 index was 10%. The total score is 143 by drawing lines, which correlates with a 74.2%, 58.9%, and 56.5% probability of being recurrence-free at 3-, 4-, and 5-year follow-up.

**Figure 3:**
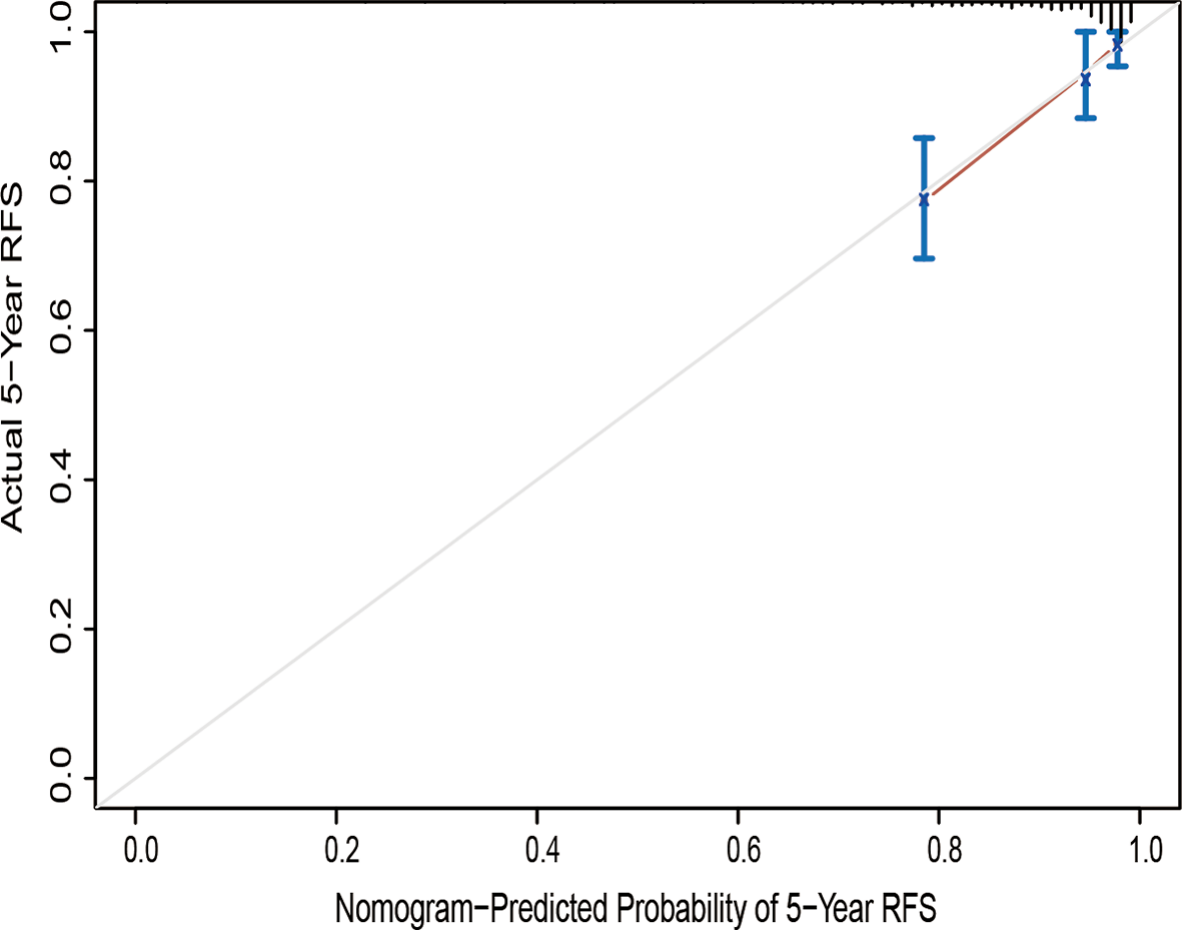
Calibration curve of the nomogram of the model prediction and the observed recurrence-free survival. RFS, recurrence-free survival.

**Figure 4:**
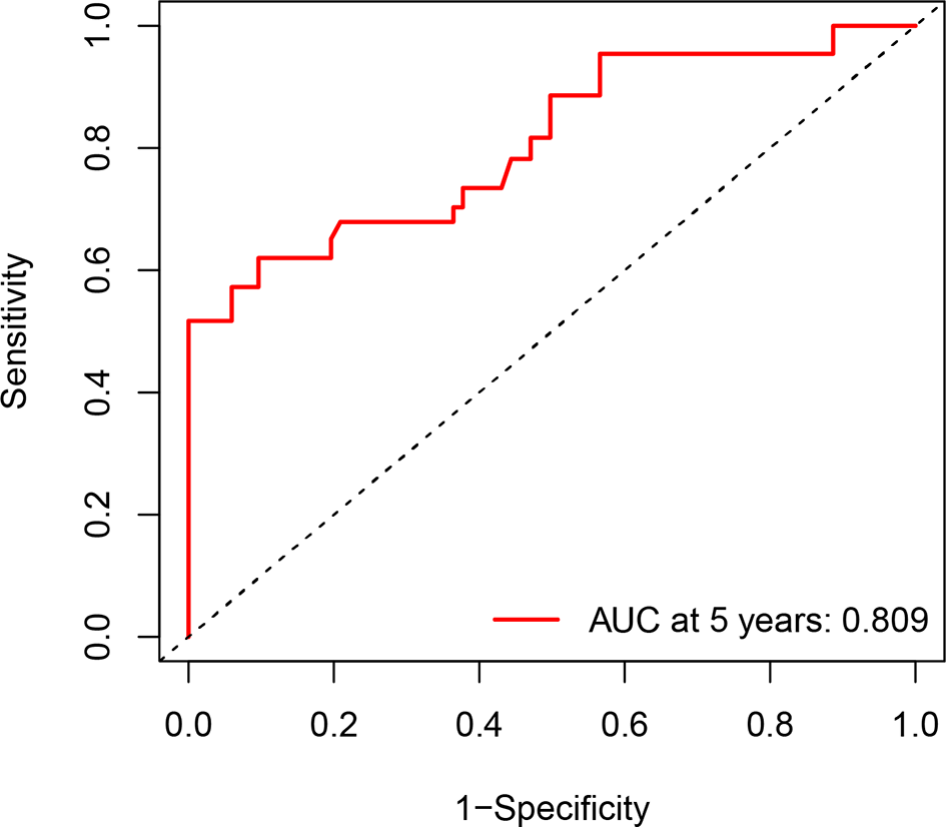
Receiver operator characteristic curve for the 5-year recurrence-free survival. AUC, areas under the curve.

The calibration curve for the probability of progression-free survival showed that the nomogram prediction had good consistency with the actual observation at 5 years (**Figure 3**). The AUC of the nomogram for predicting RFS within 5-year is shown in **Figure 4**. The AUC of 5-year was 0.809, indicating that the model had favorable discrimination.

**Figure 5:**
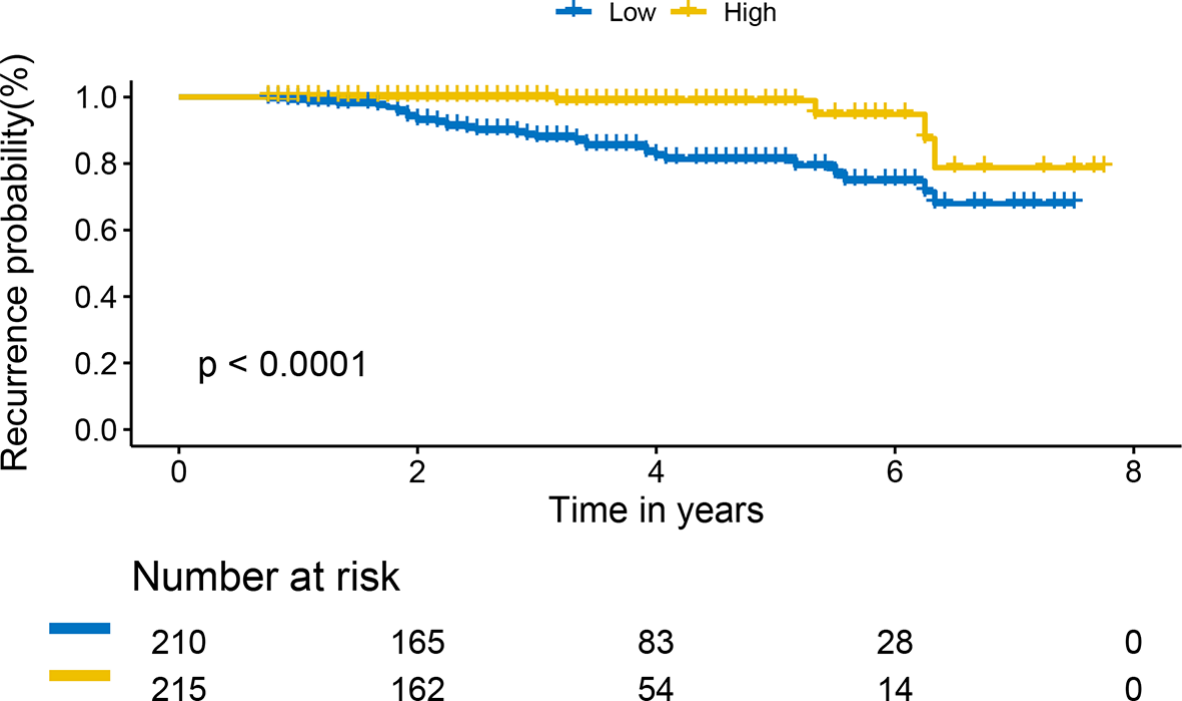
Kaplan-Meier curves of recurrence-free survival for patients based on the nomogram risk grouping.

We used the R package of nomogram Formula to calculate the nomogram score of each patient, stratified it by a median, we divided all patients into two risk groups: ‘low risk’ (<55), and ‘high risk’ (>=55). The KM survival curves showed that the nomogram risk grouping had satisfactory discrimination for RFS (**Figure 5**)

**Figure 6:**
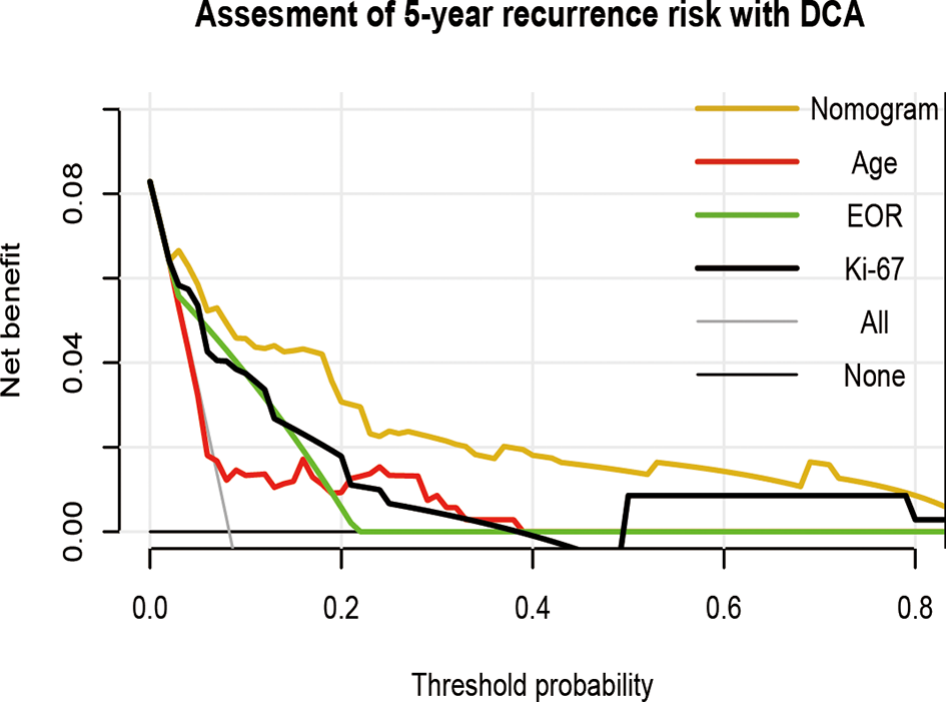
Decision curve analysis of the nomogram in predicting RFS of patients with vestibular schwannoma. EOR, extent of resection.

To visually show the clinical benefits of the nomogram, we performed a decision curve analysis DCA. The results showed that the nomogram had a positive net benefit to patients, and compared with a single factor evaluation system, the nomogram model has greater clinical net benefits and has obvious advantages in prognostic evaluation (**Figure 6**).

Based on the constructed nomogram model, we have generated a web calculator (https://abc123-123.shinyapps.io/VS-RFS/) for calculating the risk of postoperative recurrence of patients, which can provide convenient and fast risk assessment.

## Discussion

It is necessary to predict the recurrence risk of patients with vestibular schwannoma, which can further help neurosurgeons identify patients with a high risk of recurrence after VS surgery, to develop personalized follow-up and treatment strategies for VS patients. However, there is a lack of relevant forecasting tools. In this study, age, EOR, and Ki-67 were identified as independent risk factors for postoperative recurrence of VS by COX regression analysis. Subsequently, these three predictors were merged into a nomogram to calculate the risk probability of recurrence tailored to individual patients. To facilitate the use in clinical work, we also generate a user-friendly graphical interface, which will help clinical doctors to identify patients with a high risk of recurrence and personalized health care decisions.

Mehdi et al. ‘s algorithm based on an artificial neural network used clinical data of patients to predict postoperative recurrence (11). In this model, the researchers included EOR, follow-up time, and patient symptom characteristics as risk factors, showing good performance. However, considering the instability of patients’ symptoms after surgery, this model may be more suitable for predicting patients’ clinical manifestations during follow-up. In addition, follow-up time is a risk factor for recurrence, and it is also an important outcome that clinicians pay attention to. Therefore, we use the risk proportional model that takes the follow-up time into account for modeling, which enables us to estimate the patient’s risk of recurrence at a specific time. We noticed that this study also used the time interval after treatment and EOR to build a model based on the logistic regression algorithm, but it did not show acceptable performance (AUC = 0.64). In our study, we used data from a tertiary academic referral center and, based on COX regression analysis, re-explored the risk factors used to construct the predictive model, greatly improving the model’s effectiveness (C-index = 0.86). Calibration curves and decision curves showed that the model had good accuracy and clinical benefits.

EOR is a strong predictor for postoperative recurrence of VS, with patients who underwent STR having a nearly 11-fold greater risk of recurrence than the patients treated with GTR, and the estimated recurrence-free survival rates at 5, 10, and 15 years following STR were 47%, 17%, and 8%, respectively (15). Jeffrey et al. reported a 13.6% recurrence rate after STR at a median of 41.0 months (16). Jonathan et al. reported a 30% recurrence rate after STR over an average follow up of 3.1 years (17), and measured the volume of residual tumor after STR, they found that residual tumor volume and residual tumor in the internal canal were closely related to the recurrence of vestibular schwannoma (17). It is important to note that patients with GTR are still at risk for recurrence. In Hirofumi et al. ‘s case series, 52 of 396 patients relapsed after surgery with a median follow-up of 7.5 years, and the estimated 5, 10, 15, and 20-year relapse-free survival rates after GTR were 96%, 82%, 73%, and 56%, respectively. They also found that the interval between treatment and recurrence was generally longer following GTR than after STR, highlighting the importance of risk prediction and risk stratification.

More attention should be paid to pathological features. Ki-67, a nuclear marker associated with tumor cell proliferation, is associated with the progression, metastasis, and prognosis of various tumors (18). Recent research evidence suggests that Ki-67 may be closely associated with the recurrence of VS. In the study conducted by Prueter et al., patients in the elevated Ki-67 group had over a 40 times chance of experiencing a recurrence or regrowth compared to those in the low Ki-67 group (10). In a retrospective cohort study by Manas et al., it was found that when Ki-67 was used as a single predictor, an AUC of 0.735 was achieved (9), and the existence of Ki-67 improved the efficiency of the nomogram.

Young patients with VS are rare, only 1.98% were under the age of 21 (19). However, studies have shown that vestibular schwannomas in young patients are more aggressive, have higher vascular characteristics, and tendency to relapse (20). Our study also showed that younger patients had a relatively high risk of recurrence. In a retrospective cohort study of 596 patients conducted by Nick et al., the risk of tumor recurrence was significantly higher in younger patients than in older patients and suggested that this may be due to some host-related factor that promotes tumor growth (7). Luciano et al. found that in younger patients, tumor adhesions were more common and tended to be larger in size, abundant in blood supply, and excessive intraoperative bleeding (21). This suggests that vestibular schwannomas in young patients seem to have special characteristics, which should be paid attention to during the treatment. Close postoperative neuroimaging follow-up may help in the timely detection of recurrent tumors.

Studies have suggested that tumor size may be related to postoperative recurrence, because tumor remnants are prone to occur during the process of resection of large tumors and these tumor remnants may lead to recurrence (22). Besides, Raja et al. conducted a systematic review of the literature and pointed out that patients with recurrent vestibular schwannomas may have relatively large primary tumors (23). However, the current statistical evidence supporting the correlation between tumor size and tumor recurrence is still insufficient. In the study of Hirofumi et al., data from 414 patients with vestibular schwannoma with a median follow-up of 6.9 years were analyzed, by univariate COX regression analysis, they found no increased risk of recurrence in the larger tumor group compared to the smaller tumor group (6), which was consistent with our findings in this study. Recent studies have identified that tumor remnant was a risk factor for tumor recurrence (6, 17), thus, direct measurement of residual tumor volume and assessment of tumor histopathology may be more appropriate to predict tumor recurrence risk.

Although the nomogram has achieved a good degree of discrimination, calibration, and clinical benefits, we believe that it still needs to be improved in the future; The first is to include the volume of the residual tumor as a risk factor in patients with partial tumor resection to achieve a more personalized risk prediction (17). This requires the use of standardized volume measurement software and methods to reduce measurement errors of different institutions; The second is to include more detailed pathological classification because the recurrence rate of cellular schwannomas is much higher than that of classic schwannomas (24, 25). However, this distinction has not received extensive attention for a long time. The density of macrophages is one of the predictors of tumor regrowth after STR, which may improve the performance of the model to a certain extent (26). In addition, this study also has some limitations, mainly based on the nature of the retrospective study, missing data and bias are inevitable, and the predictive power of nomograms needs to be confirmed and optimized in a multicenter prospective cohort. The short follow-up time was a further limitation of this study. Control of benign tumors may fail in the long term. For prediction of long-term (>5 years) outcomes, our model may underestimate the risk of recurrence. Therefore, our model may be more accurate for identifying high-risk populations for short-term recurrence after surgery. Finally, we must remind that although the nomogram based on multiple indicators has the advantages of convenient use and high accuracy, the appropriate patients must be carefully selected when applying this nomogram to avoid erroneous estimates, for example, we excluded patients with NF2, patients with recurrent vestibular schwannomas, and patients who had previously received radiation therapy from our study population. Because of the specificity of the population, we thought it might be more appropriate to develop separate predictive models for these subgroups.

## Conclusions

We developed a nomogram that has high accuracy in predicting RFS in patients after resection of VS. All of the included prognostic factors are easy to obtain. The nomogram can improve the postoperative management of patients and assist clinicians in individualized clinical treatment. We have also generated a web-based calculator to facilitate clinical application: https://abc123-123.shinyapps.io/VS-RFS/.

## Data Availability Statement

The original contributions presented in the study are included in the article/**Supplementary Material**. Further inquiries can be directed to the corresponding author.

## Author Contributions

ZZ performed the formal analysis, contributed to the methodology, administered the project, and wrote the original draft of the manuscript. DZ curated the data and collected the resources. XS conceived the study and wrote and reviewed the manuscript. BT and YL curated the data. JZ supervised the study and wrote, reviewed, and edited the manuscript. All authors contributed to the article and approved the submitted version.

## Conflict of Interest

The authors declare that the research was conducted in the absence of any commercial or financial relationships that could be construed as a potential conflict of interest.

## Publisher’s Note

All claims expressed in this article are solely those of the authors and do not necessarily represent those of their affiliated organizations, or those of the publisher, the editors and the reviewers. Any product that may be evaluated in this article, or claim that may be made by its manufacturer, is not guaranteed or endorsed by the publisher.
