# Supplementary material for: A Nomogram to Predict Recurrence-Free Survival Following Surgery for Vestibular Schwannoma
Source: Front Oncol. 2022 Apr 28;12:838112. doi: 10.3389/fonc.2022.838112 (PMC9097914; doi:10.3389/fonc.2022.838112)
Supplement: Supplementary file 1 [file Table_1.docx]

Supplementary Table 1 Comparison of demographic information between the regular follow-up cohort and lost to follow-up cohort

|  | Regular follow-up cohort | Lost to follow-up cohort | *p* value |
| --- | --- | --- | --- |
| N | 425 | 142 |  |
| Age^#^ | 48.5 (12.1) | 49.8 (12.2) | 0.294 |
| Sex* |  |  | 0.638 |
| Female | 249 (58.6%) | 80 (56.3%) |  |
| Male | 176 (41.4%) | 62 (43.7%) |  |
| BMI^#^ | 24.4 (3.8) | 24.6 (3.4) | 0.650 |
| Side* |  |  | 0.182 |
| Left | 204 (48.0%) | 59 (41.5%) |  |
| Right | 221 (52.0%) | 83 (58.5%) |  |
| Tumor size^#^ | 2.9 (1.0) | 3.0 (1.1) | 0.269 |
| EOR* |  |  | 0.188 |
| GTR | 283 (66.6%) | 103 (72.5%) |  |
| STR | 142 (33.4%) | 39 (27.5%) |  |
| Ki-67^†^ | 5.0 (2.0-5.0) | 3.0 (2.0-5.0) | 0.608 |

* Data were expressed as number (%);

^#^Data were expressed as the means (± standard deviations);

^†^Data were expressed as the medians (interquartile ranges);

EOR: extent of resection.
